# Comparing simulation and physical kilobots performance in two ant-inspired foraging behaviours

**DOI:** 10.3389/frobt.2026.1828495

**Published:** 2026-09-28

**Authors:** Niamh Ellis, Jonathan Kelly, Alexander Caravaggio, Ian Young, Fabio Manfredini, Maria Elena Giannaccini

**Affiliations:** 1 School of Engineering, University of Aberdeen, Elphinstone, United Kingdom; 2 School of Mathematics & Computer Sciences, Heriot-Watt University, Edinburgh, United Kingdom; 3 School of Biological Sciences, University of Aberdeen, Aberdeen, United Kingdom; 4 Departement of Food, Environmental and Nutritional Sciences, University of Milan, Milan, Italy; 5 School of Computer Sciences, University of Nottingham, Nottingham, United Kingdom

**Keywords:** swarm robotics, tandem running, lone scout, robot simulator, kilobots

## Abstract

Robotics looks to nature for inspiration to perform effectively in unstructured environments and can be used to test biological hypotheses. Social animals often share information about food source locations like tandem running in ants, where a leader guides a naive recruit to a known profitable food site. This is extremely advantageous as it allows the sharing of important information among colony members, but it also has costs, such as waiting time inside the nest when no leaders are around, and a reduced walking speed for the tandem couple compared to individuals. Whether and when these costs outweigh the benefits is not well understood as it is challenging to study complex social behaviours in nature. We developed two kilobot-based approaches to compare tandem running and lone scout foraging (using an isotropic random walk) where an ant searches for food without any previous knowledge of its location: one approach based on real-life experiments and the other one on computer simulations. We tested whether the simulation-based comparison of the two bio-inspired strategies reliably predict the performance in the real-life scenario and we investigated the role that the size of the search arena played in the effectiveness of foraging. Tandem pairs discovered the food source faster in real-life experiments; however, this result was reversed in the simulations. These results highlight the inconsistencies between simulation and real-life experiments, previously reported for other systems and known as reality gap, and show how the use of simulations can lead to qualitatively different conclusions about the relative effectiveness of bio-inspired foraging strategies.

## Introduction

The robotics industry has been fast developing, with a large number of robots being currently employed in areas such as manufacturing (Cellan-Jones, 2019), healthcare (Greig et al., 2025) or the military: the number of applications is expected to continue its growth as the technology develops. This growth has brought the need to design robots that can function in unstructured and chaotic environments, very different from the highly structured environments that robots have typically been built for (Thorpe and Durrant-Whyte, 2003). Designing and programming a robot that can respond to the vast and unpredictable possibilities of unstructured environments is extremely challenging. To overcome these challenges researchers have often looked to the natural world to understand how the creatures within behave and have used this as an inspiration for robots’ behaviour. In this work, we took inspiration from ants’ foraging behaviour in order to inform robot navigation strategies when robot searchers do not know the location of the target position. Specifically, we compare the outcome of two strategies, a random search, lone scout foraging, and one where one individual guides another to a known destination, tandem running, with the aim to test whether simulation can reliably predict the relative performance of competing bio-inspired foraging strategies when transferred to physical robot swarms. While several studies modeled tandem running in ants using simulation (Goy et al., 2021), (Abramov et al., 2024a), (Abramov et al., 2024b) ours is the first to do so using a real-life robotic platform and to compare the two strategies in real-life robot experiments.

As well as using bionspiration to inform robotics, robots can be used as models of animal systems to test hypotheses regarding their behaviour (Webb, 2000; Auke, 2014). A multitude of problems can be solved using a number of simple agents working in parallel rather than a single complex agent: this is identified as swarm robotics (Brambilla et al., 2013). Robot swarms are cheap, re-configurable and function as simple modular units that can tackle a task as a team: for these reasons, swarm robotics has been identified as one of the current high impact challenges in robotics (Yang et al., 2018). One area of research that has received much attention lately is the use of robot swarm to simulate social behaviour, i.e., the complex network of interactions that characterize coordinated and cooperative work observed in many animal groups or societies, including humans (Bredeche and Fontbonne, 2022). Systems that implement swarm intelligence possess key properties such as self-organization, scalability, and robustness (Cianci et al., 2006). The potential applications for swarm robotics are endless, from the medical field with microrobotic swarms that can significantly increase dose delivery (Yu et al., 2018), to helping humans in social tasks such as brainstorming (Alhafnawi et al., 2022), and underwater exploration (Berlinger et al., 2021). However, it has often proven challenging to predict how robotic swarms behave in an experimental setup using simulations, a step that is needed before robotics can be implemented in real-life scenarios: this discrepancy is often referred to as “reality gap” (Jakobi et al., 1995). While it has been suggested that experiments with real-life robots can be avoided if using a different model for the simulated behaviour (Ligot and Birattari, 2018) (Ligot and Birattari, 2020), this approach might miss important aspects of a real-life scenario, for example, calibration is needed for the kilobots to walk in a straight line due to the irregularities in the motor vibration output and in the physical structure that influence the dynamics of physical interactions of the kilobots with the substrate where they operate. While this reality gap has been thoroughly documented for swarm robotics (Christiano et al., 2016), (Meier et al., 2021), (Zhong et al., 2018) there are no works, to our knowledge, that explored the reality gap using kilobots to compare two different strategies of foraging behaviour.

Kilobots are swarm robots designed by Harvard with each kilobot having a diameter of 3.3 cm (Rubenstein et al., 2014a). Kilobots communicate across a short range using infrared and are designed to work collaboratively (Prasath Kumar et al., 2021); nevertheless, each kilobot can be assigned a unique ID, which makes this platform suitable for testing scenarios where individual kilobots are allocated different tasks and cooperate to achieve a common goal. Some of the best examples of cooperative behaviour among animals come from the world of social insects ((Robinson and Barker, 2017), (Rodrigues et al., 2022)) and previous research has successfully employed kilobots to simulate collective behaviour in ants (Rubenstein et al., 2014b). In one of these studies, a simple algorithm was designed to encompass searching behaviour by foraging kilobots to identify suitable food sources, returning to the nest to inform other nestmates about the new food source, leading them to the new food and finally bringing the food back to the colony (Otsuka et al., 2017). The study provided an excellent first validation of the possibility of effectively simulating ant foraging behaviour and recruitment of nestmates using kilobots. Other studies that were published shortly after used swarm robotics to assess ant foraging, with a particular focus on the optimal distribution for collective foraging systems (Talamali et al., 2020) or the effect of colony size on recruitment strategy (Mayya et al., 2019). All these studies highlight one of the main advantages of using robot swarms in the context of cooperative behaviour: the benefit of a strategy can be evaluated at the level of the individual as well as at the group or colony level, therefore accurately reflecting the ecological framework where the “superorganism” behaves (Kennedy et al., 2017). The premise of our study, instead, was based on previous work on the adaptive value of tandem communication in ants (Goy et al., 2021).

Ants are social insects that are able to communicate about profitable food and nesting sites with nestmates (Robinson et al., 2008). One way that ants have to recruit nestmates to a known site is tandem-pair running, a strategy that involves a pair of individuals, where one leads the other in pursuit of a goal, usually food or a new nesting site (Franklin, 2014). This strategy is often used in the context of foraging and allows for the follower to be taught how to collect food items like seeds or to hunt (Franks and Richardson, 2006). Cooperation between the two members of the pair allows them to survive against other threats or to hunt more successfully. The behaviour has been seen in insect colonies and in some birds (Folk, 1992). Despite its benefits, tandem running in ants is not as widespread as one would think, being mainly restricted to smaller colonies (Grüter et al., 2018). In a large colony, in fact, broadcasting information to a large number of ants at once via chemical communication (pheromones) is more efficient (Sophie et al., 2008), and has been shown to improve foraging success in simulated swarms (Hecker et al., 2012). In addition, a colony may not use tandem-pair foraging for other reasons. It requires a series of cognitive tasks, which start with the leader that has to learn and memorize visual landmarks along the path that connects the food site to the nest, and continue with a form of teaching from leader to follower (Hoppitt et al., 2008), (Sasaki et al., 2020): this translates into the time that it takes for recruitment within the nest, and the lower rate of travel that tandem pairs exhibit. Furthermore, for all these reasons, tandem running is more complex and slower than solo scouting, the more typical foraging strategy where a single and naive ant explores the surroundings for food (Franks and Richardson, 2006), and failure of the tandem pair results in more time lost that could be spent on foraging. In an observation of tandem-pair running (Grüter et al., 2018) it was found that of 138 tandem runs 106 (76.8%) were successful, while unsuccessful runs were caused by interference from vegetation, other ants, or the leader leaving the follower behind. From an engineering point of view, these two modalities of foraging in ants, each with its pro and cons, represent a powerful benchmark to test what factors influence strategy efficiency because (1) proof-of-concept is already provided by the biological system, (2) coordination of several physical tasks is needed for success, and (3) optimal behaviour requires cooperation among robots (Alan, 2009).

The question as to where the use of tandem running in ants is worth the extra resources that are required for its implementation was first explored by Goy et al. (Goy et al., 2021) using an agent-based simulation approach. In that study, the proposed model predicted that the spatial-temporal distribution of resources, colony size and the ratio of scouts and recruits all have a substantial impact on the success of resource procurement. The results suggested that in low resource environments, with large margins in quality and resource life-time, tandem running is favoured. In our study, we moved one step further in the comparative analysis of two foraging strategies observed in ants, namely, lone scouting and tandem-running recruitment - lone scout being the behaviour where single ants explore the environment on their own to find new food sources (Hölldobler and Wilson, 1990). This was achieved by implementing a series of experiments with physical (real-life) swarm robots, that were then compared with computer simulations. Past studies, in fact, have revealed how the direct comparison between real-life experiments with kilobots and simulation presents numerous issues, contributing to the reality gap challenge (Meier et al., 2021) (Christiano et al., 2016). These studies combined the use of real robots and simulations to reproduce both stochastic and deterministic foraging behaviours (Qi et al., 2019). Our work is in line with this field of research, in that we used ant foraging behaviour to compare the basis for a stochastic (lone scout) vs. a deterministic (tandem running) behavioural process (Goy et al., 2021).

In our experimental design we included the use of a series of landmark beacons during tandem running trails, simulating a series of visual cues that form a memory path in the brain of the leader of the pair–while for the follower these are simple stimuli that can be perceived and incorporated. These landmarks are broadly used in nature by many ant species as they guide their movements towards a previously discovered food source or to the nest (Bühlmann et al., 2011), (Cheng et al., 2012). The beacons are necessary as the lead robot in the tandem pair does not know where the food is but only knows to follow the series of beacons and stop when it finds the food. In swarm robotics the use of stationary beacon robots that can communicate with other robots in their neighbourhood has been used to simulate pheromone trails ((Brian Werger and Mataric, 1996); (Payton et al., 2001); (Nouyan et al., 2009)), another common foraging strategy adopted by ants that relies on chemical cues (Sophie et al., 2008). The main advantage of this approach is that the system can be implemented with simple robots in unstructured environments; the main disadvantage is that the beacon robots are not contributing to foraging (Talamali et al., 2020). Furthermore, in our trials, we tested the effect of experimental arenas of different sizes (to reflect the variability of foraging range in the natural environment) on the success of the different foraging strategies, both with real kilobots and in simulation. Experiments were conducted in the robotics laboratory at the University of Aberdeen (see Figure 1) while the simulations were performed using the software CoppeliaSim. We chose to use CoppeliaSim as it has a large number of useful features, including its suitability for high precision modelling and video recording (Pitonakova et al., 2018), fewer errors and overall better rating compared to other simulators (Farley et al., 2022) and is capable of modeling multiple robots simultaneously while including an extensive library of standard mobile robot models (Collins et al., 2021). It is also noted that CoppeliaSim supports multiple scripting languages providing flexibility in writing code to control robots and simulation environments (Tseeva et al., 2024).

**FIGURE 1:**
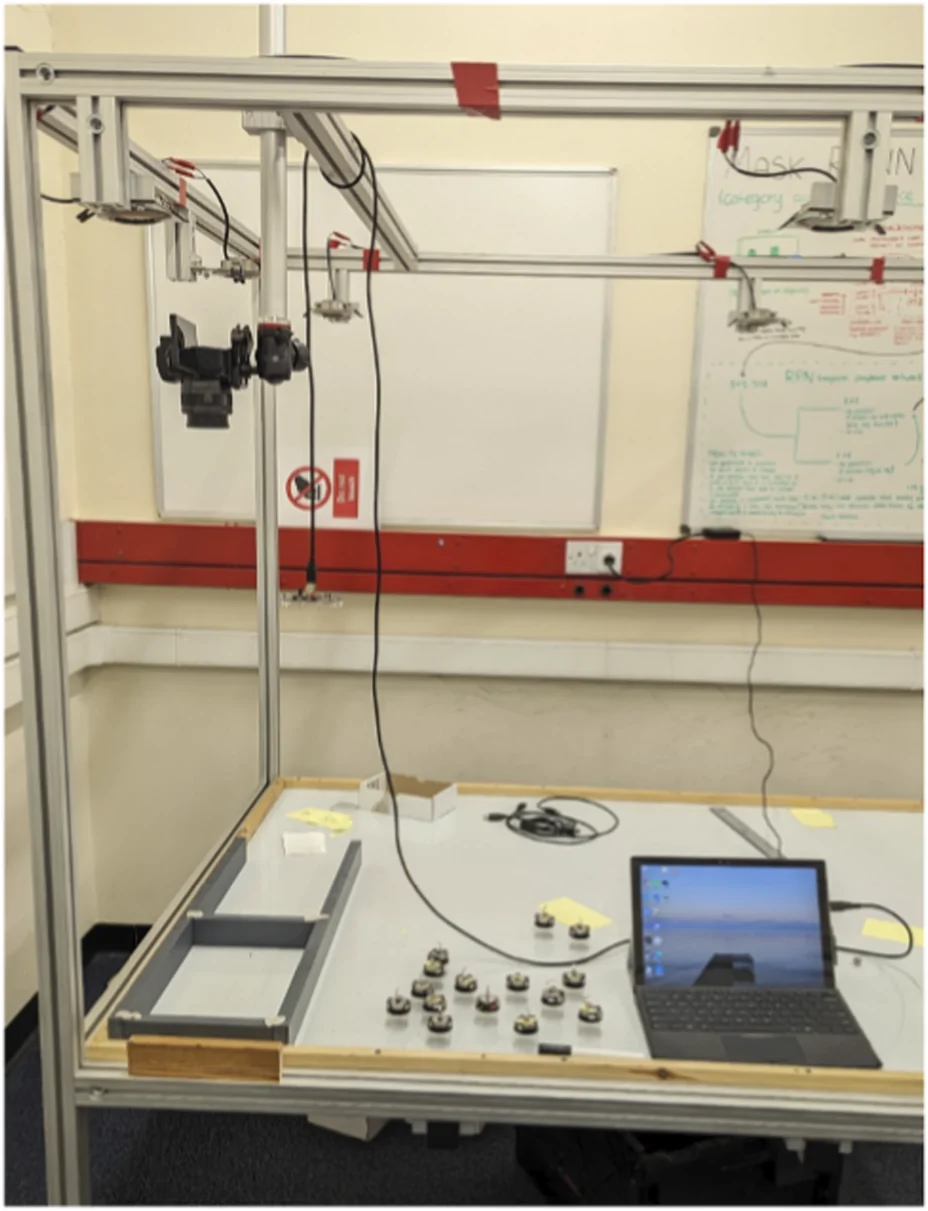
Experimental arena for Kilobots with overhead camera, kilobots, controller and laptop.

This study makes two contributions. First, we provide a controlled comparison of two contrasting ant-inspired foraging strategies in both simulation and physical Kilobot swarms. Second, we demonstrate that the simulation-to-reality discrepancy can extend beyond differences in absolute performance to produce different relative rankings of competing strategies.

## Methods

### Laboratory experiments with real-life kilobots

An arena was used to perform experiments with real-life kilobots in the lab. The arena was built on a tabletop area bounded by planks of PVC, to adjust the area size as needed. To avoid the planks being nudged by the kilobots they were held in place by adhesive putty. The experiments were recorded with a Canon EOS M50 camera placed above the experimental area as seen in Figure 1. The videos were stored and the time stamps of the videos were used to determine the foraging duration. This was measured from the exact time when the kilobots started moving to the moment when the follower (in the case of a tandem pair) or the lone scout found the food. The kilobots LEDs were used to easily discern the kilobots state for accurate time keeping. The LED flashed purple before a test had commenced, displayed a solid colour during the tests, depending on the kilobots direction of movement, and turned white when the test was complete. Experiments began with a set up of kilobots reproducing the behaviour of a tandem pair (leader and follower, see Figure 2). Stationary kilobots known as beacons were positioned in a line in order to simulate a series of visual landmarks recreating a memory path that guided the leader to the known food site. Kilobots do not have independent localisation capabilities, so these beacons are needed to guide them along the memory path. Each beacon transmitted a unique ID and the leader in the tandem pair was constantly searching for a lower ID than the one being received - with food source having the lowest ID (ID 0) while every beacon in the trail had an ID that was one measure higher than the previous: the follower was in direct contact with the leader and followed its movements. Therefore, for a set-up with two beacons and a food source, the first beacon in the trail would have ID 2, while the beacon next to the food source would then have ID 1. This resulted in the tandem pair (i.e., the leader followed by the follower) orbiting around each beacon in turn until the next beacon was detected - at which point the pair began orbiting around it. This design allowed for more beacons to be added for further trials. The follower always kept behind the leader and both communicated with the beacons and the food kilobot - when it was reached. All these kilobots were positioned at 8 cm apart from each other, measured from the centre of each kilobots, with the exception of the follower that was 6 cm from the leader. Marks on the whiteboard surface of the area were used to keep track of the initial positions and orientations of the kilobots. Once the ID for the food source was found (ID 0) the tandem leader continued its orbit until the follower was within a set distance (4.5 cm) of the food source. At which point it was considered that the follower had found the food source and the time was recorded.

**FIGURE 2:**
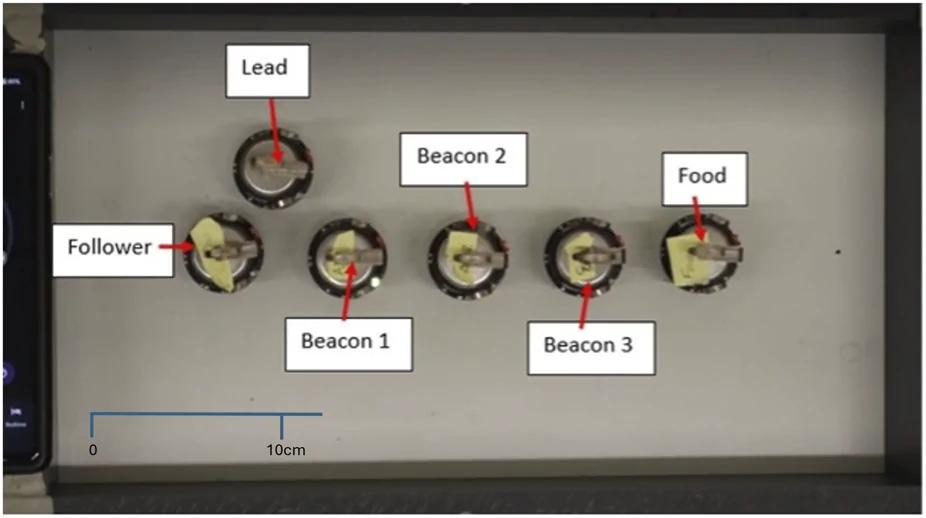
Kilobots in tandem pair configuration: the “lead” kilobot guides the “follower” using the stationary “beacons” as a guide to the “food”. Kilobots are 3.3 cm in diameter (Rubenstein et al., 2014a).

During a tandem run, the two members of the pair aimed to maintain a close distance between each other. The follower started orbiting around the beacons when it detected a distance greater than 5 cm between itself and its leader. The leader moved when it detected a distance lower than the same threshold between itself and the follower. We are aware that these dynamics do not fully reflect what observed for tandem pairs of ants in nature, where interruptions often occur as well as breakups - as we report in the Introduction and Discussion sections. However, the logistics of working with kilobots forced us to keep the system simple to make it possible for the tandem pair to reach the food site: in fact, our main goal (and the real novelty of this study) was to test the efficiency of tandem pair behaviour in different scenarios and not to assess the implementation of this behaviour with kilobots *per se* - something that has been achieved by previous research as we report in the Introduction. The orbiting functionality was implemented using set distances to control the movement of the kilobot. When a beacon was detected as being less than 3.3 cm away the kilobot moved forward. When it was between 3.3 cm and 4.5 cm away the kilobot instead turned left, if the distance was greater than 4.5 cm then the kilobot turned right. We tested three different scenarios, each one with a different number of beacons, corresponding to different lengths of the memory path: one, two and three beacons (Figure 2). A width of 19 cm was chosen for the arena to ensure that the tandem pair had sufficient area to move around the beacons. The arena length instead changed according to the number of beacons used, as it had to accommodate not only the beacons themselves but also the space in between them. The smallest arena length was 25.5 cm (one beacon), after which the arena lengths were increased by 5 cm each time. Table 1 shows the arena size for each of the tests and the number of beacons in each arena size.

**TABLE 1:** Arena sizes and number of beacons for real-life kilobot experiments.

Arena length (cm)	Arena size (cm^2^)	Number of beacons
25.5	484.5	1
30.5	579.5	2
35.5	674.5	3

The second part of the experiment involved using individual kilobots that behaved like scouts foraging for food alone. The lone scout kilobot simulated an ant scout searching for a source of food without prior knowledge of its location in the area around the nest, hence without relying on a memory path. The lone scout (see Figure 3) used therefore a random search to locate profitable food sites. This was implemented by programming the lone scout kilobot to turn towards a random direction (either clockwise or anticlockwise) for 2.5 s and then move straight for 5 s before turning in a random direction again. The implemented behaviour is an isotropic random walk, which has equal probability of moving left or right (Dimidov et al., 2016) and was chosen for its simplicity. The parameters for the random search (2.5 s for turning and 5 s for moving straight) were chosen to allow effective exploration of the surroundings. In order for the kilobot to fulfil its role as a lone scout and find the food source, it must be able to explore the whole arena. Hence, while the kilobot must be able to turn in a different direction, it must not turn fully back on itself. 2.5 s were found following empirical observation to be the optimal time to reliably guarantee a change of direction. Pilot trials were also conducted with the values for turning and going straight being the same (5 s): however, in this case, the kilobot turned too far and did not produce a random search behaviour. In addition, 5 s of forward movement was found following empirical observation to be enough time to thoroughly explore the arena and not such a long time that the lone scout would quickly reach the boundaries of the arena.

**FIGURE 3:**
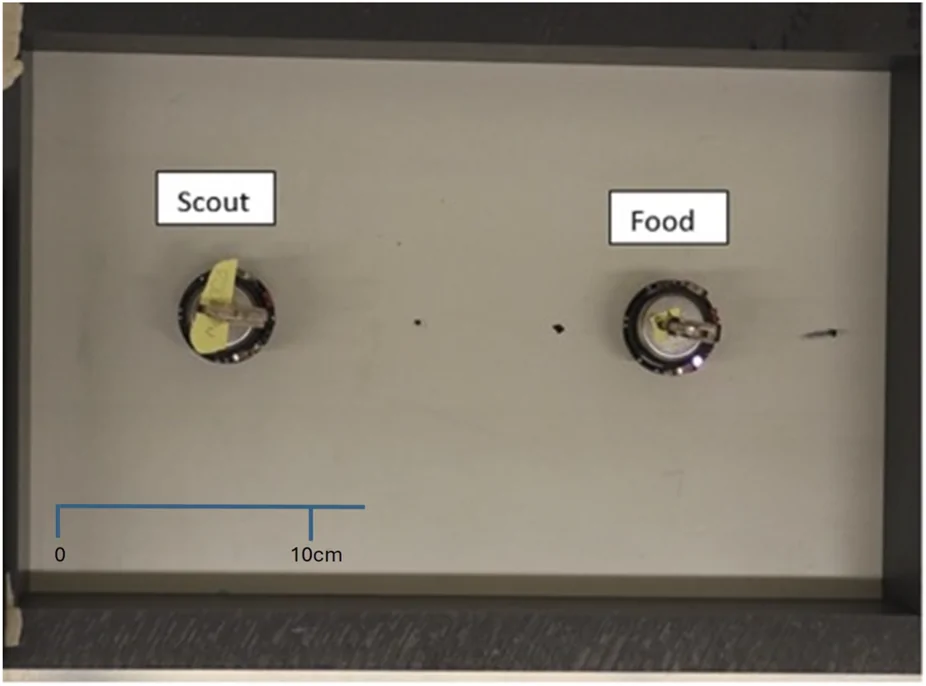
Kilobots in lone scout configuration, with lone scout (left) and food source (right).

This random search behaviour continued until the scout came within the same set distance of the food source as described for the tandem pair. At this point the time was recorded and the experiment concluded. At the beginning of the trial, the lone scout was placed in the same position as the follower kilobot in the tandem pair as described above and the food source maintained the same position. This was done to keep the foraging distances the same for both experiments.

All kilobots were controlled and calibrated during the experiment using kilogui (Julien Tharin, 2016), a software that uploads programs to the kilobots via the overhead controller. Once the custom hex code has been uploaded, commands can be sent to the kilobots to run, stop, or reset the currently uploaded program. Due to strong inter-individual variations, to achieve reliable locomotion, each robot must be calibrated individually to determine suitable motor parameter values that enable straight movement and rotations at the desired speed (Pinciroli et al., 2018) Calibration was done on each moving kilobot using the kilogui software. To calibrate the left turn a value is selected for the right motor, then varied until until the robot can perform a full turn consistently. The process is repeated for the right turn. To calibrate to go straight, the values already found for turning left and turning right are used as an initial guess. Adjustments can be made by use slightly lower values to fine-tune straight movement (Labs, 2026). The tandem pair experiments were repeated five times following previous work in the same area (Valentini et al., 2018), (March-Pons et al., 2024). For the lone scout we increased the sample size to 15 given the high standard deviation that we observed in the fist trials.

### Simulations with CoppeliaSim

A simulation of the aforementioned robot foraging strategies was created with the purpose of checking how transferable the observations with real-life kilobots would be in a virtual environment, more suitable for testing additional experimental conditions like, for example, a wider range of arena sizes. The functional CoppeliaSim kilobot model developed by K-Team (Julien Tharin, 2016) was utilised in the simulations with Engine Bullet 2.78: this model can simulate a multi-agent kilobot system, with capabilities that can simulate if not mimic directly the communication and locomotion of ants. We utilise the Bullet 2.78 physics engine as it has previously been found to increase the performance of our chosen simulation software (Pinciroli et al., 2018) and as it produced the least number of issues (Farley et al., 2022).

All the behaviours designed for experiments with real Kilobots were reproduced as closely as possible in the simulation software CoppeliaSim (Coppeliasim robotics, 2023) so that the outputs of the two approaches could be directly compared. For the tandem running behaviour, the same number of beacons and the same leader/follower interactions were replicated in the simulation. For the tandem pairs, we also utilised the same distance metrics as in real-life experiments: kilobots designated as beacons or food source were stationary and set with a unique ID corresponding to their position in the trail (Figure 4). The two members of the tandem pair were also set up as in the experiments with real Kilobots, with each member orbiting around the beacons on the way to the food source. A few modifications were introduced in the code with respect to the experiment with real-life kilobots. The distances used in the orbiting function were altered so that the kilobot moved straight when sensing a beacon that was less than 3 cm away, turned left when the beacon was between 3 cm and 5 cm away and turned right when the beacon was greater than 5 cm away. It was found that these distances were better for maintaining the orbiting behaviour and not interfering with the beacon trail. The lone scout behaviours was also mimicking very closely the real-life experiments, with a kilobot randomly exploring the arena in search of the only other kilobot - representing the food. The code for the lone scouts was also slightly modified. As stated previously, when using real-life kilobots, the lone scout turned for 2.5 s and moved straight for 5 s. The simulated kilobots, on the other hand, turned and moved straight for the same period of time (5 s). In fact, during the initial testing, it was determined that this caused the simulation runs to fail less frequently. A simulation run was considered to have failed when a kilobot became stuck in a corner and after attempting to go straight toppled over. At this point the kilobot is on its side instead of its legs and hence cannot continue its exploration of the arena. Kilobots were more likely to become stuck at the wall and hence end up in the corner when the turning value was 2.5 s as opposed to 5 s. This was observed in 10 simulation runs in the 579.5 cm^2^ arena size. This behaviour was only observed in simulation and a video showing this behaviour has been added to the supplementary material in the Zenodo repository. Simulations were run for the same arena sizes as for experiments with real-life kilobots, as shown in Table 1 and in Figure 5.

**FIGURE 4:**
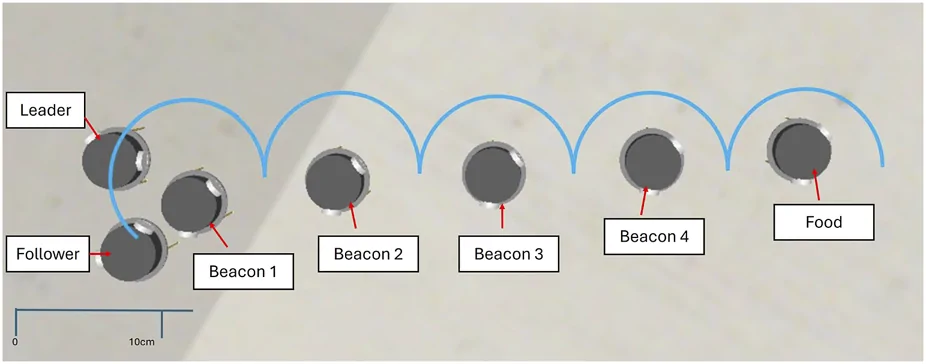
The path taken by the tandem pair kilobots following three “beacon” kilobots to the object “food” kilobot - extreme right. The overall trajectory is straight, which is compatible with ants’ behaviour when the food site is known and they are guided by landmarks (Hunt et al., 2018).

**FIGURE 5:**
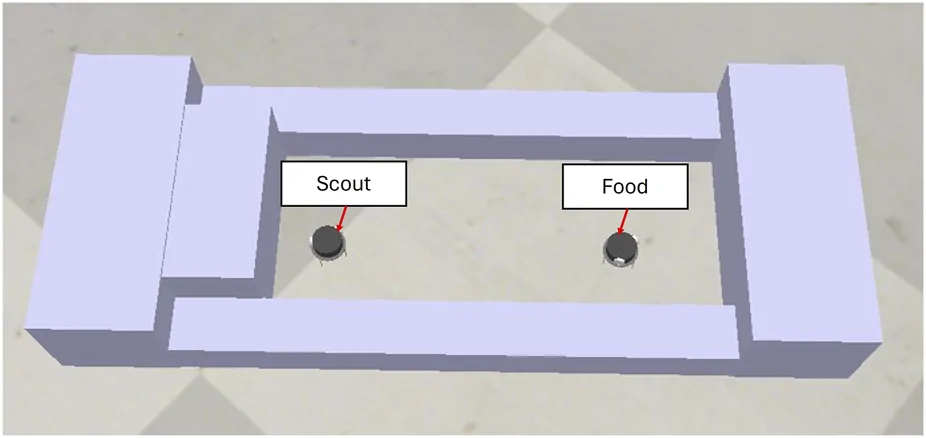
Arena for kilobots in simulation, with lone scout (left) and food source (right).

### Combinations

In a tandem pair of foraging ants, the leader already possesses the information on where the food source is located, and acquiring that information takes time. In a set of additional tests, we decided therefore to incorporate the time required by a leader to find a new food source and added it to the time used to recruit a follower to the site. To do so, we used data obtained from the assays described above to combine a lone scout run with a tandem pair run, producing all possible combinations: this was done for both experiments with real-life kilobots and simulations, for all arena sizes. Analogously, we produced all possible combinations of two runs performed by lone scouts, so that the two foraging techniques could be directly compared in a more realistic setup. The total number of combination was calculated using a binomial coefficient function implemented in MATLAB called “nchoosek”: this function produced a total of 3003 possible unique combinations of all lone scout and tandem pair runs, for each arena size, that were used for both real-life kilobots and simulations. This approach had two additional benefits: it allowed including the time it takes for the leader of the pair to find the food source in the initial exploratory trip (hence making the strategy more similar to what observed in nature) and it also mitigated against the extremely high variance that was observed in the duration of individual runs performed by lone scouts, both in the assays with real kilobots and in the simulations.

### Statistical analyses

We used SPSS Statistics software to analyse data. We first assessed the distribution of data and checked whether they met the requirement for the application of parametric statistics via the Shapiro-Wilk test. Since there was evidence of non-normal distribution of data, we decided to apply non-parametric statistics (Mann-Whitney U tests) to assess whether the duration of runs for tandem pairs was significantly different from random searches of lone scouts for each arena size. In all comparisons, a threshold of α=0.05 was used to invoke statistical significance. We also calculated confidence intervals for all observations: these are included in the Supplementary Materials (Supplementary Table S1).

## Results

Experiments with real-life kilobots revealed that the duration of the search to find the “food” beacon between tandem pairs and lone scouts was very similar in the arena with the smallest size (484.5 cm^2^, Table 2): this difference was not significant (z = −0.22, p = 0.827). Starting from the arena of intermediate size (579.5 cm^2^) a trend was observed for tandem pair strategy to become faster than lone scout strategy, though the difference was not significant (z = −0.31, p = 0.760). The trials in the largest arena (674.5 cm^2^) revealed the most evident difference in duration, with tandem pairs finding the food site faster than lone scouts, although analysis showed no significant results (z = −0.65, p = 0.513). The range of effect size - in absolute values - for this set of experiments was 0.15 (small arena), 0.36 (medium arena) and 0.60 (large arena).

**TABLE 2:** Summary of the results obtained in the experiments with real-life kilobots and simulations. The median times (sec) for each category of kilobot experiment are shown in the table.

Experiment type	Foraging behaviour	Arena length		
		25.5 cm	30.5 cm	35.5 cm
Real-life	Tandem pair	41.60 s	72.50 s	120.30 s
	Lone scout	42.10 s	77.00 s	156.60 s
Simulation	Tandem pair	158.21 s	268.01 s	317.05 s
	Lone scout	111.70 s	165.61 s	259.01 s

Simulations with CoppeliaSim revealed an opposite trend: tandem pairs took longer than lone scouts to find the food source for all arena sizes, and the difference was most visible in the arena of largest size (Table 2). In this case, effect size - in absolute values - ranged from 0.01 (small arena), to 0.21 (medium arena) and 0.33 (large arena). None of the comparisons produced statistically significant results: this was the case for small (z = −0.57, p = 0.570), medium (z = −1.53, p = 0.127) and large size (z = −1.00, p = 0.315) arenas. It is interesting to note that the average time to find the food for tandem pairs was approximately three times higher in the simulations than in the experiments with real kilobots.

We performed an analysis to provide a quantitative measure of the gap between simulation and real-life observations, calculated by subtracting the value (in seconds) of measures in real-life from simulations (Table 3). This analysis showed that overall the gap increased as arena size increased but this pattern was more regular in the lone scout strategy, where it went from 69.6 s (small size arena) to 88.61 s (medium size arena) and 102.41 s (large size arena); in the tandem pair strategy instead, the gap started higher (116.61 s in the small arena), jumped to 195.51 s in the medium size arena and stayed pretty much stable in the large size arena (196.75 s). Interestingly, when measuring standard deviations and standard errors associated with the duration of trails in simulation vs. real-life, the difference between measures for simulation and real life (in absolute value) was small for tandem pair experiment (between 0.99 and 14.32 for standard deviation; between 0.44 and 6.41 for standard error) while for lone scout trials the gaps were much higher and showed a progressive reduction from small to medium and large arena size (from 99.55 to 9.32 for standard deviation; from 25.74 to 2.41 for standard error). In Table 3 we have also provided a measure of percentage deviation for each arena size as an additional normalised metrics to quantify reality gap.

**TABLE 3:** Quantification of the gap between simulations with Coppelia SIM and real-life kilobot assays. For each one of the three arena sizes (small, medium and large) we calculated the difference between the median times (in seconds) employed by the kilobots to reach the food source–simulation minus real-time observation (Gap). We also obtained differences between standard deviation and standard error measures obtain for the two scenarios (expressed in absolute values; St dev and St err, respectively) and percentage deviation (% dev) measures. We performed this set of analyses for the two foraging strategies (tandem pair and lone scout).

Arena Size	Tandem pair				Lone scout			
	Gap	St dev	St err	% dev	Gap	St dev	St err	% dev
SMALL	116.61	1.03	0.46	116.72	69.60	99.55	25.74	90.51
MEDIUM	195.51	14.32	6.41	114.83	88.61	74.82	19.32	73.05
LARGE	196.75	0.99	0.44	89.97	102.41	9.32	2.41	49.28

Combination tests produced very similar outputs to what observed in data from the initial experiments. The difference in searching times for a tandem pair + a random search by a lone scout vs. two random searches by lone scouts was minimal for the smallest arenas, while it became progressively larger for the arenas of intermediate and largest size (Figures 6, 7; Table 4). As observed for the initial experiments, tandem pair strategy became progressively faster than lone scout search for real-life kilobots as arena size increased, while in simulations lone scout strategy became faster. Differently from the output of the initial experiments, all comparisons for real kilobot combinations were statistically different (p < 0.001) while in the case of simulations, only the arenas of intermediate and largest size showed significantly different search times (p < 0.001) while this was not the case for the arena of smallest size (p = 0.53).

**FIGURE 6:**
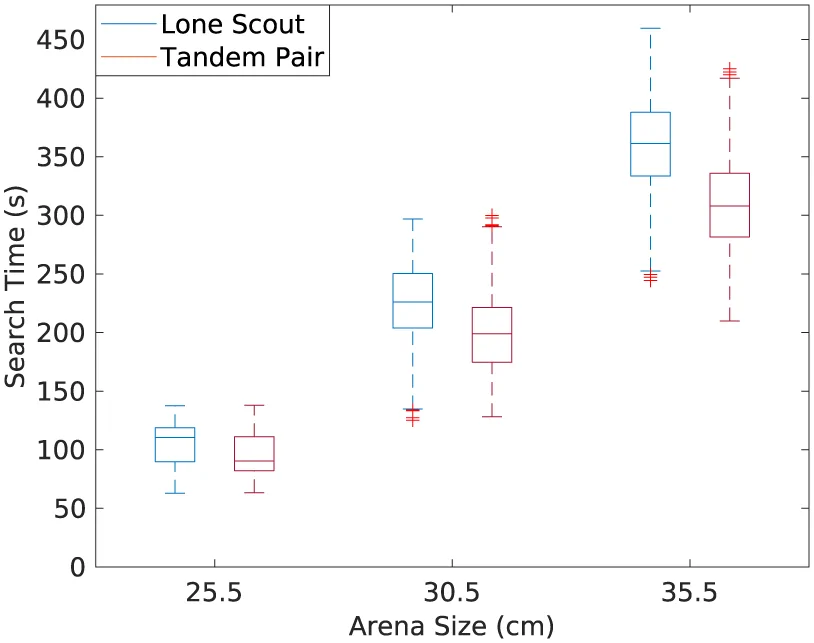
Boxplot visualization of the time elapsed for the combinations of the real-life experiments, for each arena size. In blue, “Lone Scout” = combinations of all possible lone scout + lone scout runs. In red, “Tandem Pair” = combinations of all possible lone scout + tandem pair runs. Boxes indicate median (central line) plus the 1st and 3rd quartile, while whiskers show minimum and maximum values.

**FIGURE 7:**
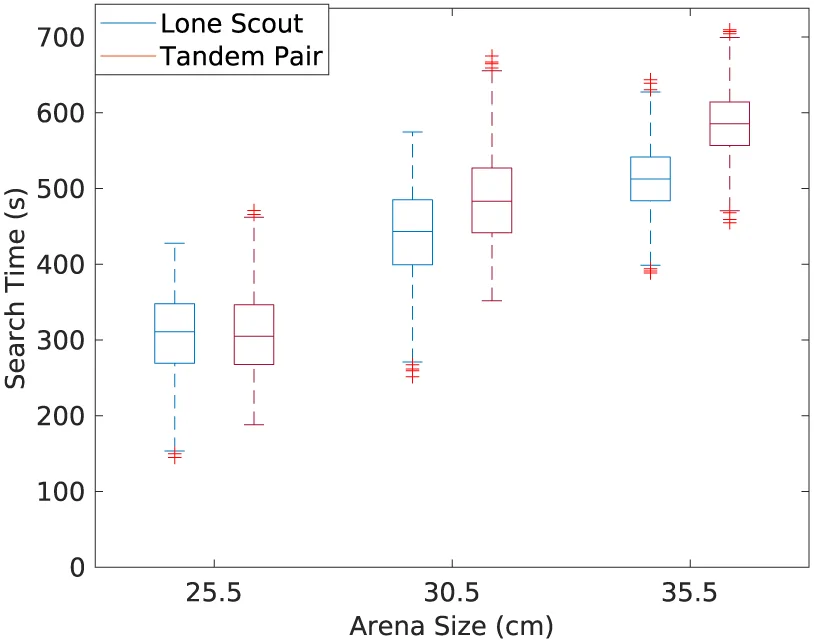
Boxplot visualization of the time elapsed for the combinations of the simulation experiments in CoppeliaSim, for each arena size. In blue, “Lone Scout” = combinations of all possible lone scout + lone scout runs. In red, “Tandem Pair” = combinations of all possible lone scout + tandem pair runs. Boxes indicate median (central line) plus the 1st and 3rd quartile, while whiskers show minimum and maximum values.

**TABLE 4:** Summary of the results obtained in combination tests. For each arena size the median times (sec) are shown.

Experiment type	Foraging behaviours	Arena length		
		25.5 cm	30.5 cm	35.5 cm
Real-life	Lone scout + tandem pair	90.40 s	199.04 s	308.06 s
	Lone scout + lone scout	110.50 s	226.00 s	361.40 s
Simulation	Lone scout + tandem pair	304.90 s	483.35 s	585.69 s
	Lone scout + lone scout	310.82 s	443.18 s	512.62 s

## Discussion

In this study we reproduced two foraging strategies that are typical in ant colonies: tandem pair recruitment and lone scout foraging using kilobots, both in real life and simulation. We compared the performance of the two strategies in each environment, to assess which behaviour would lead to the food source in the shortest amount of time: tandem pair foraging was quicker for real-life kilobots, while in simulations lone scout foraging performed better. The main novelty of this study is the demonstration that simulation-to-reality discrepancies can alter the relative ranking of competing bio-inspired behaviours and consequently lead to different conclusions about which strategy should be selected for physical deployment.

Our results show that both for the real-life kilobots and simulation, there was no difference in time needed to reach the food between tandem pairs and lone scouts for the smallest arenas (though time spent in simulations was visibly higher than with real-life kilobots) while differences between the two strategies started to emerge from intermediate arenas and became more prominent for the arenas of largest sizes - i.e., when the food source was the farthest from the foraging ants. These differences followed opposite trends in the two approaches and, while in the real-life experiments and simulation they were not statistically significant, all but the arena of smallest size show statistically significant results in the combinations. It is possible that the lack of statistical significance for this set of experiments could be due to the limited sample size that we used: this can be improved in the future by increasing the number of replicates tested in each set of analyses. The aforementioned observations were confirmed when performing combination tests to control for initial search time of leaders in tandem pairs and for the high variation observed across individual random searches: in this case a statistical significance was observed for arenas of intermediate and large size.

Our results also highlight how difficult it is to use simulations to model the behaviour of kilobots in real life - something that has been reported before in the literature (Meier et al., 2021), (Zhong et al., 2018). We discuss these results in the context of ant foraging behaviour as well as in relation to the reality gap in robotics and other applications of kilobots and robotics to biological scenarios.

In Goy et al. the authors found that the time needed for recruits and scouts (equivalent to our tandem pairs and lone scouts, respectively) to find the food was highly affected by the combination of conditions in the experimental environment, namely, colony size, quantity and quality of the food and distance from the nest. In our study, the number of variables assessed was limited, as we wanted to focus on the comparison between experiments with real-life kilobots vs. simulations: we tested increasing arena sizes (hence distance of the food from the colony), while we had only one food item of high quality. Therefore, a direct comparison with Goy et al. is not straightforward but it is still noteworthy that our experiment with real-life kilobots aligned well with what was reported by the authors for limited numbers of high quality food sources: in this scenario, tandem running is faster than the random search of a lone scout. It will be interesting to assess in the future the output of experiments with real kilobots when adding more variables, such as, for example, more food items of different value. Alternatively, some existing variables could be changed, for example, replacing the simple isotropic random walk with Levy-flight movements, which have been shown to provide superior area coverage for swarm foraging (Schroeder et al., 2017).

As for the results of our simulations, the inconsistency with real-life kilobots and simulations performed by Goy et al. shows the aforementioned reality gap. To better understand the origin of this reality gap, it is useful to distinguish between epistemic and aleatoric sources of uncertainty. Epistemic uncertainty arises from imperfect knowledge of the robot and environment (e.g., friction and contact dynamics), whereas aleatoric uncertainty is intrinsic to stochastic decision-making. The two foraging algorithms presented in the current study incorporate different levels of stochasticity. By design, in the tandem running algorithm stochasticity is present at a much reduced level compared to the lone scout algorithm. The higher stochasticity of lone scout strategy is confirmed by the higher standard deviation consistently observed for this group in all arenas (see Supplementary Figure S1 in the supplementary material). When comparing the measure of reality gap (simulation minus real-time observation) between the two algorithms (Table 3) we see that it varies more in the less stochastic algorithm–associated with tandem pair strategy - compared to the more stochastic–lone scout. From this we deduce that stochastic decision-making is not the main source of the reality gap: instead, this is more likely being caused by epistemic uncertainty, for example, the accuracy of dynamic models used in the simulators.

A few possible issues that affect the accuracy of dynamic models can be considered. As the simulated world is a combination of ideal conditions there is no effect of friction acting on the kilobots in the random search as there is in real life. The surface the kilobots interact with in simulation is perfectly smooth, while real life surfaces have small imperfections (nicks and bumps) in which the kilobots legs could get stuck. Additionally, kilobot legs can be bent, affecting not only the speed of the kilobot but how it turns. Real-life kilobots need charging while simulated ones do not. This means that the behaviour of a real-life kilobot can degrade over time, while the behaviour of a simulated kilobot remains consistent. Real-life kilobots require calibration while simulated kilobots do not. Hence, simulated kilobots will always move in a perfectly straight line, provided that both motors are programmed with the same values, which is not necessarily the case for real-life kilobots. Finally, real-life kilobots rely on the surface being reflective enough to communicate, while in simulation this is based on proximity. This could affect communication timings and hence alter behaviour.

CoppeliaSim was chosen as the simulation environment due to its various physics engines and more efficient CPU use, compared to other simulators (Pitonakova et al., 2018). This study clearly show that the CoppeliaSim platform is not able to accurately simulate the complexity of real-life experiments with kilobots. One of the most striking examples of this discrepancy was observed for the orbiting functionality, which was not performed by the kilobots in the real experiment as it was in the simulation (Zhong et al., 2018). Simulations are fundamental to inform planning experiments with real kilobots but, at the same time, the dynamic models used in them cannot fully capture the complexity of the environment a robot is acting in - which is nevertheless significantly simpler than the real life where organisms live and behave. Even in the over-simplified foraging-arena of a robotic lab there are many factors that influence dynamic models, such as individual mass or collisions and traction among robots (Christiano et al., 2016) that might have an influence on the outcome of the robot-robot interactions, but are only partially considered by the simulation software (Meier et al., 2021). Some help could come from more advanced physics engines, the programs that simulate physics phenomena, or from a more carefully selection among the physics engines available in a simulator. A recent study, in fact, reported that Open Dynamics Engine or ODE provides results that are the closest to the output of a real-life environment among all tested physics engines (Meier et al., 2021).

When it comes to assessing our results in the context of foraging behaviour in ant colonies similar considerations as above still hold: in fact, previous studies reported that tandem followers are faster than lone scouts to find a single food source (Franks and Richardson, 2006). This is corroborated by swarm robotics research showing that recruitment significantly increased foraging efficiency compared to experiments where robots could not communicate (Krieger et al., 2000). It was also found that ants were more likely to perform tandem runs and they travelled faster when the food source was more distant (Grüter et al., 2018) - similar to our arenas of increasing size. In this last study, however, the authors also report that tandem running has costs, mainly associated with the logistic challenges of two ants moving together in a complex environment, characterized by a wide range of obstacles such as impervious substrates and predators (just to mention a few), and with the possibility of breakouts in the tandem pair. For example, the previous literature reported that in different species of ants, individual foragers can be 1.5 to 4 times faster foraging than tandem pairs (Schultheiss et al., 2015), (Franks and Richardson, 2006); furthermore, a breakup of the tandem-pair was reported 32 times out of 138 observed runs (or 23.2%) in (Grüter et al., 2018). In a separate set of experiments with real kilobots, we observed that three out of the eight tests with tandem pairs failed, which means a failure rate of 37.5% (*unpublished data*). This might have been caused by localisation errors, for example, the malfunctioning of the kilobots’ infrared proximity sensors. This seems to be a much higher rate than what observed in nature, but we must take into account that the sample size in our experiments was limited and this might have influenced the outcome. Using augmented reality for Kilobots (ARK) (Reina et al., 2017), (Talamali et al., 2020), which provides Kilobots with extra information based on their location and state, could reduce the errors that occurred during our experiments and likely caused the occurrence of the reported breakouts.

Our study introduces an innovative approach in the comparative assessment of complex animal behaviours using kilobots: however, it only represents a first step that paves the way to a range of possible implementations in the future, aimed not only to producing a more robust system, but also one that more closely resembles the reality of the behaviour of interest. One obvious development of this research will be to test whether the same patterns that we observed (i.e., faster foraging times for tandem-pair strategy compared to lone scouts) hold for arena of larger sizes. In our experiment we stopped at three beacons due to the high number of failures observed when including four beacons but further experiments could use a round arena (with smaller likelihood of kilobots getting stuck in a corner) or multiple food kilobots around the perimeter of the arena. It would be more convenient to test this in simulations of course, as less challenging logistically; however, our findings implicate that this will not be enough and experiments with real kilobots will also be needed. Secondly, a limitation of our experiments is that we did not exploit the full potential of the kilobots platform, designed so that a large number of agents can act in parallel as a swarm: this was motivated by the need to keep the experimental design simple, being this the first series of trials of this kind. In the future it will be interesting to exploit the ability of swarm robotics to successfully carry out a task by using a high number of agents. This will translate into multiple kilobot ants performing the food search in parallel, adopting both tandem pair and lone scout strategies, which more closely reflects the dynamics occurring within an ant colony. In addition, the light sensor on board the kilobots could be used for phototaxis (Rubenstein et al., 2014a) as an alternative to following the beacon trail in the implementation of tandem running. While light following is highly bioinspired, implementing phototaxis reliably to follow a trail would be more challenging than using a beacon trail so the latter version was selected for the initial implementation. A limitation of this study is that it does not incorporate experiments involving multiple food sources, barriers along foraging routes, or dynamically changing environments. Since such complexities are inherent to real-world ant foraging systems, investigating them would provide a valuable avenue for future research.

## Conclusion

In this study, we successfully simulated two ant foraging behaviours, tandem running and lone scouts, in a controlled environment, establishing a platform that can be used in the future to study and simulate other complex animal behaviours. Our results demonstrate that, in the foraging behaviours and robotic platform investigated here, simulation and physical experiments can produce different relative rankings of competing strategies. The results also show that the time required to complete the task differs between the two strategies only for bigger arenas. Immediate application of our findings could be, for example, in the selection of robot navigation strategies when the desired target location is unknown and in the fields of swarm intelligence, collective decision making and navigation techniques and strategies. The potential application of these findings to more distant fields such as logistics, agriculture, and search and rescue highlights the importance of this research and its findings.

## Data Availability

The datasets presented in this study can be found in online repositories. The names of the repository/repositories and accession number(s) can be found below: https://zenodo.org/records/13825200.
