# Supplementary material for: Comparing simulation and physical kilobots performance in two ant-inspired foraging behaviours
Source: Front Robot AI. 2026 Sep 28;13:1828495. doi: 10.3389/frobt.2026.1828495 (PMC13654705; doi:10.3389/frobt.2026.1828495)
Supplement: Supplementary file 1 [file Supplementaryfile1.docx]

**Supplementary Material for Ellis et al.**

**“Comparing simulation and physical kilobots performance in two ant-inspired foraging behaviours”**

**Table S1**

Shown in the table are the 95% confidence intervals calculated for real-life kilobots and simulations tested in both tandem pair and lone scout foraging modalities for the three arena sizes.

|  |  | **SMALL ARENA** | | **MEDIUM ARENA** | | **LARGE ARENA** | |
| --- | --- | --- | --- | --- | --- | --- | --- |
|  |  | **Lower bound** | **Upper bound** | **Lower bound** | **Upper bound** | **Lower bound** | **Upper bound** |
| **real-life kilobots** | **tandem pair** | 32.105 | 53.535 | 57.651 | 115.669 | 101.046 | 157.834 |
|  | **lone scout** | 27.048 | 78.325 | 63.978 | 161.609 | 122.287 | 237.727 |
| **simulations** | **tandem pair** | 143.594 | 167.596 | 253.965 | 276.409 | 301.902 | 356.241 |
|  | **lone scout** | 72.605 | 234.145 | 130.199 | 310.699 | 193.530 | 319.294 |

**Figure S1**

Variation in standard deviation measures obtained from recordings of time spent to find food (expressed in seconds) for the two foraging strategies - Tandem pair and Lone scouts. Comparisons are visualized for the three arenas of different size: small (top panel), medium (middle panel) and large (lower panel). Lone scouts show consistently higher standard deviation which is in line with the higher stochasticity associated with this foraging strategy.
